# Factors to consider before choosing EV labeling method for fluorescence-based techniques

**DOI:** 10.3389/fbioe.2024.1479516

**Published:** 2024-09-18

**Authors:** Magdalena Dlugolecka, Malgorzata Czystowska-Kuzmicz

**Affiliations:** Chair and Department of Biochemistry, Medical University of Warsaw, Warsaw, Poland

**Keywords:** extracellular vesicles, f-NTA, nanoscale flow cytometry, fluorescent staining, corona, lipophilic dyes, lipoproteins

## Abstract

A well-designed fluorescence-based analysis of extracellular vesicles (EV) can provide insights into the size, morphology, and biological function of EVs, which can be used in medical applications. Fluorescent nanoparticle tracking analysis with appropriate controls can provide reliable data for size and concentration measurements, while nanoscale flow cytometry is the most appropriate tool for characterizing molecular cargoes. Label selection is a crucial element in all fluorescence methods. The most comprehensive data can be obtained if several labeling approaches for a given marker are used, as they would provide complementary information about EV populations and interactions with the cells. In all EV-related experiments, the influence of lipoproteins and protein corona on the results should be considered. By reviewing and considering all the factors affecting EV labeling methods used in fluorescence-based techniques, we can assert that the data will provide as accurate as possible information about true EV biology and offer precise, clinically applicable information for future EV-based diagnostic or therapeutic applications.

## 1 Introduction

Small extracellular vesicles (EVs) are sized 30–150 nm and play a significant role in cell-to-cell communication because they are secreted by all eukaryotic cells and carry a specific cargo of lipids, nucleic acids, and proteins derived from the origin cell (Colombo et al., 2014). They are present in all body fluids and can be easily obtained by minimally invasive methods. Because EVs contain molecular cargo similar to that of the parent cells, they seem to be a promising source of biomarkers as so called “liquid biopsy” (Imanbekova et al., 2022).

Direct measurement of sEVs using conventional flow cytometry is impossible because their size is below the limit of detection of the instruments (Arraud et al., 2016; Botha et al., 2021; van der Pol et al., 2022). Moreover, their small size also has some implications in the way we can label EVs and how labeling impacts the measurement. Often, there is no possibility of washing the unbound dye/antibody; we can only dilute it, but it is still present and may interfere with results.

**FIGURE 1:**
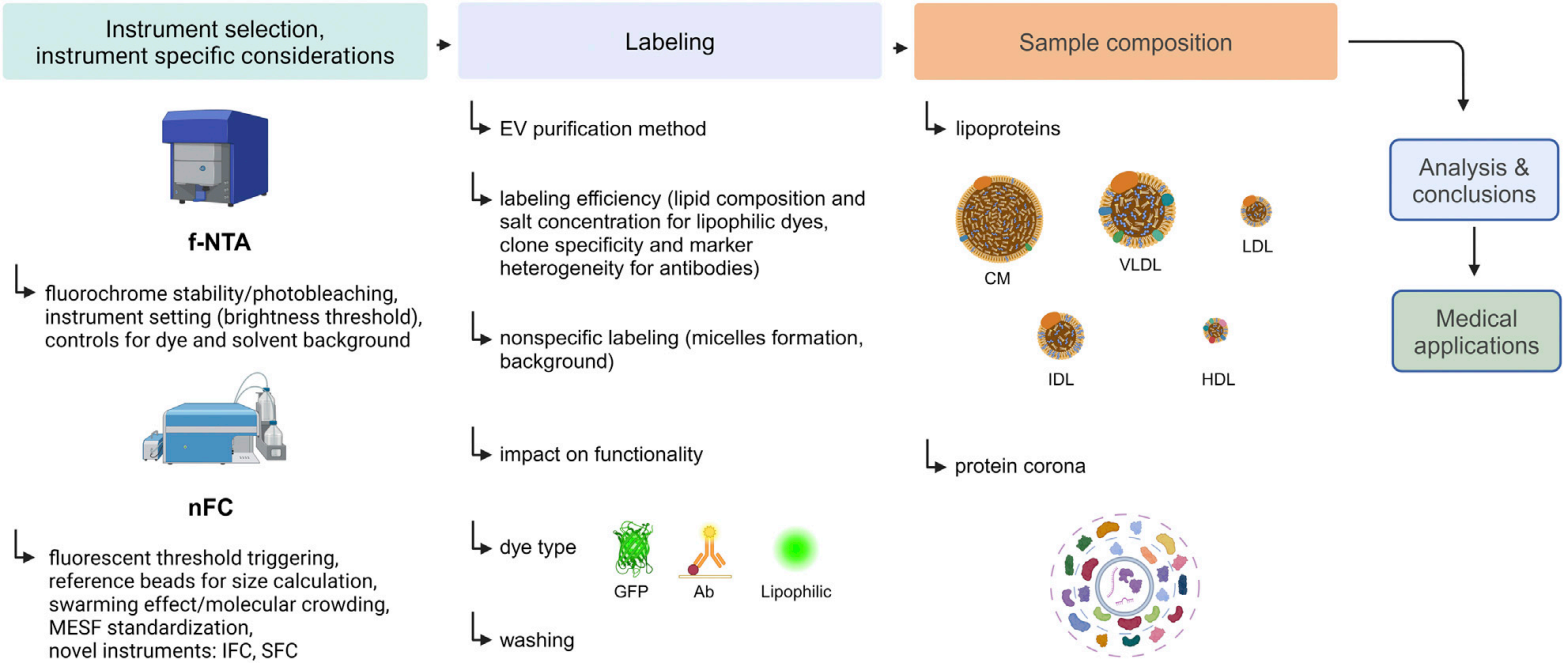
An experimental flow chart of an exemplary fluorescence-based analysis of EVs. Created with BioRender.com.

To address these challenges, several highly sensitive instruments capable of directly measuring fluorescently labeled small EVs have been developed. Methods on which the performance of these instruments is based, with the focus on fluorescence-based Nanoparticle Tracking Analysis (f-NTA) and Nanoscale Flow Cytometry (nFC), are presented in this review. Subsequently, important fluorescent labeling parameters influencing EV measurement, such as labeling efficiency, specificity, and impact on EV functionality, are discussed. Next, factors that may interfere with EV analysis, such as lipoproteins and the protein corona (PC) will be considered. Finally, mistakes commonly made during the planning and conducting of fluorescent labeling experiments of EVs, as well as typical pitfalls and misinterpretations of results, are discussed. A flowchart presenting the steps of an exemplary fluorescence-based analysis of EVs and listing the interfering factors that should be considered is shown in Figure 1.

## 2 Fluorescent methods

The most frequently used methods capable of directly measuring fluorescently labeled particles smaller than 300 nm are f-NTA and nFC, which are discussed in more detail in this chapter. Additionally, other methods that can be applied to a limited extent for EV characterization have recently been developed, such as those based on direct stochastic optical reconstruction (dSTORM), or a single-particle interferometric reflectance imaging sensor (SP-IRIS) coupled with fluorescence microscopy.

In brief, the dSTORM method allows for single-molecule localization super-resolution microscopy (with an optical resolution of ∼20 nm) using regular, photostable, and bright organic fluorophores (Endesfelder and Heilemann, 2015; Chambers et al., 2021; Zhang et al., 2023). Notably, Bağcı et al. described a novel approach for characterizing EVs using the dSTORM method, in which particles are immobilized on a microscope slide prior to antibody staining of specific EV proteins. They used the reflectance mode of a confocal microscope to locate the EVs plane precisely. They then identified EVs labeled with specific proteins in the fluorescence mode of confocal microscopy. This approach allowed them to distinguish labeled proteins on EVs from free proteins. The disadvantage of this method is that it requires fixation and immobilization of EVs, which affects their functionality. In addition, the true size of EVs cannot be measured and is not suitable for identifying single EVs because of the diffraction limit of confocal microscope (Bagci et al., 2022). In turn, SP-IRIS coupled with fluorescence microscopy allows multiplexed characterization and digital counting of EVs caught on a solid chip in the form of a microarray (Avci et al., 2015; Daaboul et al., 2016; Mizenko et al., 2021; Breitwieser et al., 2022).

Examples of the most common instruments with a short description of their advantages and disadvantages, along with representative references, are presented in Table 1.

**TABLE 1:** Instruments capable of direct measurement of nanosized particles with fluorescence detection.

Name of the instrument	type	company	size range of particles	cost^a^	throughput^a^	advantages	disadvantages	References
ZetaView	NTA	Particle Metrics	10 nm–2000 nm	low	high	high resolution, real-time analysis; short time is needed for sample preparation and measurements (<1 h); EVs can be analyzed in their native form in solution	limited sensitivity to smaller particles (<50 nm), biases the detection of larger particles, less precise in heterogenous samples containing differently sized vesicles, sample hast to meet the detection concentration range, possibility of co-localization studies still under development	Desgeorges et al. (2020), George et al. (2021), Bagci et al. (2022)
Nanosight (NS300, LM10, LM20, LM14)	NTA	Malvern Panalytical	10 nm–2000 nm	low	medium	EVs can be analyzed in their native form in solution, high sensitivity	requires trained personnel, careful sample preparation, limited concentration range for measurements, technical issues related to measurements of sample in flow, difficulties in determining the size of particles in heterogenous samples	Dragovic et al. (2011), Koksal et al. (2023)
Nanoimager	dSTORM	ONI	10 nm–2000 nm	medium	low	high spatial resolution, single molecule sensitivity, co-localization studies possible, intra-vesicular staining possible	limited to relatively small sample volumes, EV concentration of sample has to be known, low through-put	Roseborough et al. (2023), Zhang et al. (2023)
ExoView	SP-IRIS coupled with fluorescence microscopy	NanoView Biosciences	from 20 nm	low	medium	quick, automated platform, enables biomarker colocalization, low sample volume, no need for sample purification	limited to specific particle types – only detected by tetraspanin antibodies	Bachurski et al. (2019), Breitwieser et al. (2022), Bhagwan Valjee et al. (2024)
Cytoflex	nFC	Beckman Coulter	not specified	medium	high	high sensitivity, multi-color analysis, flexibility	variable performance depending on sample quality, high background interference, limited sensitivity to dim particles, swarming effect	George et al. (2021), Mizenko et al. (2021), Salmond et al. (2021)
Aurora/Northen Lights	nFC	Cytek Biosciences	not specified	medium	high	high resolution, high throughput, unmixing, removal of autofluorescence	high background interference, limited sensitivity to dim particles, swarming effect	Welsh et al. (2021), Voss et al. (2022), Aibaidula et al. (2023)
ImageStream	nFC	Luminex Corporation	not specified	high	high	imaging flow cytometry, multiparametric analysis direct EV measurement in biological fluids	complex data analysis using dedicated idea software, need for high quality computer equipment for data storage and analysis, complex optimalisation for every single dye or Ab	Corso et al. (2019), Tertel et al. (2020), Botha et al. (2021), Tertel et al. (2022), Wu et al. (2023)
CellStream	nFC	Merck Millipore	not specified	low	high	high sensitivity multiparametric analysis direct EV measurement in biological fluids	limited options for advanced applications, unintuitive software, need for high quality computer equipment for data storage and analysis, complex optimalisation for every single dye or Ab	Zheng et al. (2023), von Lersner et al. (2024)
nanoFCM	nFC	NanoFCM Inc	not specified	low	medium	high sensitivity, rapid analysis, colocalization analysis	limited sample throughput, accuracy relies on calibrating using silica beads causes bias	Fortunato et al. (2022), Chen et al. (2023)
MoFlo Astrios-EQ	nFC	Beckman Coulter	not specified	high	high	high sensitivity, single cell sorting	limited to specific applications, complex operation	Morales-Kastresana et al. (2017), Morales-Kastresana et al. (2019)
Apogee A50-Micro	nFC	Apogee Flow Systems	100–1000 nm	medium	high	linear detection of particles, multiplex detection	high initial investment, need for negative and positive controls, need to modify the gains for each PMT	Gomes et al. (2018), Padda et al. (2019), Botha et al. (2021), Roseborough et al. (2023)
Gallios	nFC	Beckman Coulter	from 100 to 150 nm (fluorescence threshold triggering, calculated value)	medium	high	high sensitivity compared to conventional FC, reproducible, simplicity of use, can be applied do unprocessed biological fluids	unspecific signal from unbound dye and dye aggregates, risk of swarming effect, only stained EVs visible, detection of 300 nm fluorescent beads with efficiency of 50% ± 2%, limited sensitivity of sEVs	Arraud et al. (2016), George et al. (2021)
Influx (modified for small-particle detection)	nFC	BD Biosciences	from 100 nm (fluorescence threshold triggering)	customized	high	high sensitivity, multicolor antibody labeling	unspecific signal from unbound dye and dye aggregates, risk of swarming effect, only stained EVs visible, lack of reproducibility because of custom made equipment	Nolte-'t Hoen et al. (2012), van der Vlist et al. (2012)
FACS Canto (custom constructed)	nFC	BD Biosciences	from 70 to 80 nm (fluorescence threshold triggering)	customized	high	high sensitivity	high cost for custom made equipment, unspecific signal from unbound dye and dye aggregates, risk of swarming effect, only stained EVs visible, lack of reproducibility because of custom made equipment	Stoner et al. (2016)

^a^ More details on cost and throughput are available on the website: https://exrna.org/resources/ercc2-tech-detail/?particles=&methods=.

### 2.1 Fluorescent NTA

It is well established that NTA in the scatter mode is a very sensitive method for measuring the concentration and size of particles in the nanometric size range. This method uses Brownian motion (random movement) of particles in a solution for measurement. Brownian motion is strictly related to the hydrodynamic size of particles. Because smaller particles move faster than larger ones, NTA software can calculate the hydrodynamic diameter using the Stokes-Einstein equation (Malloy and Carr, 2006).

In addition, by counting the particles in a known volume of the sample within the flow cell, the software calculates the concentration of the particles. Moreover, during NTA measurements, light is scattered by the nanoparticles, which enables their visualization using a microscope. The scattering effect depends on the refractive index of the nanoparticles and the solution in which they are dissolved. The differences in the refractive indices of the different particles enabled the differentiation of these nanoparticles in NTA. However, the value of the refractive indices significantly affects the instrument’s precision and resolution. Larger nanoparticles, which have a higher refractive index, scatter light more intensely, making them easier to detect and track. Smaller particles with a relatively small refractive index present in the same sample may not be detected or underestimated in their number, as their signal will be covered by larger particles. Populations with a close size range may not be distinguished and will be considered as one population. Therefore, the analysis of heterogeneous particle solutions, such as biofluids, with conventional scatter-based NTA may be difficult. Furthermore, if the refractive index of the nanoparticles is close to that of the solvent, they may be more difficult to detect because they scatter light less efficiently (Malloy and Carr, 2006; Filipe et al., 2010; Gardiner et al., 2014; van der Pol et al., 2014; Midtvedt et al., 2020; Kashkanova et al., 2022).

Consequently, the sensitivity and use of light scattering are limitations of conventional NTA because particulates with a similar refraction index and/or size cannot be differentiated from actual EVs. These can be dust or powder, plastic particles, lipoproteins, or other impurities. They can influence both concentration and size measurements. NTA has a strictly defined concentration range in which samples can be measured, and in most cases, the sample must be diluted to meet this concentration range. Notably, the solvent used may contain particles that are visible in the NTA, which may affect the results. Therefore, it is crucial to prepare appropriate controls, such as size and concentration calibration using commercial PS beads (with a certificate of size and concentration) and buffer-only controls (for instance, phosphate-buffered saline, PBS). In addition, one should check the quality of plastics and always use fresh deionized water or other buffers as instrument rinse solutions and sample diluents (Snyder et al., 2021). Another important requirement of NTA that ensures the accuracy and reproducibility of the readout is to maintain the same conditions for each measurement, which have been previously optimized for a given sample type (temperature, sensitivity, frame rate, threshold, etc.). These conditions should be reported when results have been published. Even if one meets all these requirements to improve reproducibility, it is important to remember that EVs are very heterogeneous and polydisperse, and their size distribution on NTA usually does not follow a Gaussian or log-normal distribution. The population of smaller EVs may be covered by a population of larger EVs with a higher refractive index, and populations in a close size range may not be distinguished and will be considered as one population.

This imperfection of the traditional light scatter-based NTA was noticed, among others, by a team investigating urinary EVs, comparing the impact of different isolation methods (different combinations of ultracentrifugation (UCF), size-exclusion chromatography, PEG precipitation and ultrafiltration) on chosen EV analysis methods (NTA, flow cytometry, transmission electron microscopy) (Droste et al., 2021). The authors showed that EV enumeration by NTA was highly affected by typical urine non-vesicular impurities like uromodulin. Another group confirmed that protein aggregates, such as albumin, which are created in urine, were visible on NTA as small particles undistinguishable from urinary EVs (McNicholas et al., 2017). Therefore, scatter-based NTA is highly dependent on the chosen EV isolation method that determines the levels of co-isolated non-vesicular impurities. This is particularly true for biological fluids, where impurities are usually highly abundant. Indeed, the impact of myosin aggregates, IgG immunoglobulins, and alpha-synuclein on NTA scatter measurements has been reported previously (Filipe et al., 2010; Hoover and Murphy, 2020).

To increase the specificity and sensitivity of NTA measurements and prevent the detection of non-EV particles, additional fluorescent labeling of particles was introduced for NTA. Fluorescent NTA (f-NTA), which measures particles in fluorescent mode, enables the visualization of only particles that are specifically fluorescently labeled. Using lipophilic or nucleic acid-specific dyes, we can detect particles that have biological membranes or DNA/RNA cargo, whereas fluorescent antibodies allow the detection of specific EV-surface antigens and phenotypic characterization. Whereas traditional light-scatter-based NTA measurements offer only an estimation of the total particle number in a solution, f-NTA enables the measurement of individual particle fractions determined through specific fluorescent labeling. Furthermore, small EVs that reflect insufficient light to be detected in scatter mode may become visible after labeling in the fluorescent mode owing to their fluorescence. Therefore, f-NTA may be a solution for the main drawbacks of conventional NTA, such as underestimation of the content of small EVs in a heterogeneous particle solution and poor distinction between “real” EVs and impurities of similar size. However, many factors that can influence readout and introduce bias remain, as outlined below.

#### 2.1.1 Fluorophore properties and instrument settings

The most well-known aspects that may affect the fluorescent staining of EVs, but also affect traditional cell analysis, are the stability and intensity of the fluorophore. In the case of f-NTA, this impact is even more pronounced, since the measurement lasts longer than on a flow cytometer; therefore, the fluorophore needs to emit fluorescence longer for signal collection and is more prone to photobleaching. Modern NTA devices are equipped with a special function (for instance, the “low bleach” option in the ZetaView device, or laser pulsing on and off in synchronization with the camera shutter in the case of the Nanosight NS300) to prevent photobleaching. To lessen the impact of photobleaching, it is important to use bright and stable fluorochromes like Alexa Fluor (488, 647), Cyanine Dyes (Cy3, Cy5) or quantum dots (Thane et al., 2019; Desgeorges et al., 2020; Fortunato et al., 2021). Although the relative high sensitivity of the NTA instruments enables to detect the much lower fluorescent signal of EVs, in comparison to cells (usually 2-3 magnitudes lower), but on the other hand it also means a higher susceptibility to background noise and contaminations (Midekessa et al., 2021). The sensitivity of the instrument for the fluorescence signal can usually be set by the brightness threshold setting. In the fluorescent mode, it affects the distribution, number, and size of the detected particles and the zeta potential (Midekessa et al., 2021). It is important to balance the dye concentration and fluorescence threshold based on appropriate controls (dye-only control and solvent-only control) before the measurement of actual samples.

### 2.2 Nanoscale flow cytometry

A noteworthy advancement in Flow Cytometry (FC) instrumentation utilized for EV analysis involves the implementation of nanoscale FC (nFC, nanoFACS). This upgrade of conventional flow cytometry includes special enhancements in optical and fluidic systems, which allows for more accurate and targeted analysis of EVs (Lian et al., 2019). In this technology a fluorescence threshold triggering instead of side scattered light (SSC) triggering is used (Gomes et al., 2018; Padda et al., 2019; Salmond et al., 2021). The wavelength of visible light is longer, which causes lower resolution, however the usage of fluorescence and a shorter wavelength – 405 nm, enables a better resolution. Simultaneously, the use of fluorescence triggering determines that, from the beginning, only distinct labeled populations of EVs are visible. The lack of a “scatter mode” in nFC differs from NTA, where the sample can be measured simultaneously in both the scatter and fluorescent modes. On the other hand, the nFC method enables often measurements directly in the original biological samples without EV isolation, which cannot be done in case of NTA because of high background (Gorgens et al., 2019). Moreover, combining nFC with size exclusion chromatography (SEC) purification after labeling enables to keep a low false positive event rate, because SEC washes unbound dye or antibody from the sample (Aibaidula et al., 2023). Thanks to nFC, characterization of the molecular EV cargo, and colocalization of different markers on single EVs is possible. However, similar to NTA, several factors must be considered for a successful nFC analysis. The importance of the specificity and effectiveness of EV labeling and the removal of unbound dye and dye aggregates are discussed in detail in the labeling section. Other factors are discussed below.

#### 2.2.1 EV size characterization using nFC

EV size characterization in scattered light using nFC remains biased because the reference beads from polystyrene and silica, which are available on the market so far, have higher refractive indices (RI) than EVs. This results in inaccurate measurements both in term of size and concentration (Gul et al., 2022). Recently a new kind of reference - hollow organosilica beads (HOBs) have been evaluated (Deumer et al., 2024). In the study authors used HOBs with different shell thickness and determined the size distribution and their concentration using several techniques including Small-Angle X-ray Scattering (SAXS), Atomic Force Microscopy (AFM) and Single Particle Inductively Coupled Plasma Mass Spectrometry (spICP-MS). They then used two different flow cytometers, A60-Micro, Apogee, and Nothern Lights, Cytek, for flow cytometry measurements and NS300, Malvern Panalytical, for NTA. The determined side scattering cross-sections in the case of HOSs were two orders of magnitude smaller than those for the PS beads but similar to that of EVs. Moreover, the measured RI value could be tuned by adjusting the shell thickness of the HOBs and for 11-HOB it was about 1.363–1.373 – which is similar to the RI of EVs in human urine. These results are promising for the future use of nFC for size measurements of EVs.

An indirect way to perform EV quantitative measurements on nFC was described by von Lersner et al., 2024. The authors used Di8-ANNEPS-stained EVs in serial dilution with addition of dextran to evaluate the advantages of the so-called molecular crowding (MC). Detecting single extracellular vesicles (EVs) in a flow cytometer often requires a significant dilution of the source material to prevent the detector from being overwhelmed by multiple particles. However, this dilution reduces the molecular density, which can increase the nonspecific interactions between microparticles and macromolecules. Therefore, the authors used dextran to compensate for the reduction in protein and other buffering components caused by the sample dilution. They found that it improved single-particle detection of labeled beads and EVs by 100%–400%. They established a 3.25% final concentration of dextran as the optimal condition for particle detection, which was also verified using synthetic beads. Moreover, in their study they developed a method named “EV Fingerprinting”, that determines separate EV populations using dimensional reduction of multiparametric data collected by nFC (von Lersner et al., 2024). This method allows to identify and characterize distinct EV populations in complex biological samples. EV fingerprinting uses multiparametric analysis of the fluorescence data of EVs stained with a lipophilic dye that is sensitive to the membrane environment. Di-8-ANEPPS changes its fluorescence properties depending on the order of the lipid membranes, allowing EVs to be distinguished based on their size and structure of their lipid membrane. Thus, it is possible to obtain more detailed information on EV heterogeneity than with traditional cytometric methods.

#### 2.2.2 Impact of the swarming effect in nFC

An important factor in nFC is the swarming effect, which means that many single particles are detected as one event by a flow cytometer (Libregts et al., 2018). When many small molecules pass through the detector simultaneously, their signals can overlap. This results in one large signal being recorded instead of several smaller ones, which can lead to incorrect conclusions regarding the size and number of measured particles and the mean fluorescent intensity (MFI) of the detected molecules. The swarming effect was detected by comparing the fluorescence intensities of different sample concentrations. If the ratio of the particle number to MFI remains constant, there is no swarming effect; however, if the intensity increases rapidly, many particles are measured together. To prevent swarming low flow rates and serial dilutions of the samples are recommended (Kuiper et al., 2021).

#### 2.2.3 MESF standardization

The fluorescent signal from the nFC is reported in arbitrary units, which cannot be compared between the instruments. To enable validation of measurements and comparison between different flow cytometers and laboratories, a so called MESF (Molecules of Equivalent Soluble Fluorophore) calibration with standard MESF beads must be performed (Schwartz et al., 2002; Padda et al., 2019; Kuiper et al., 2021; Hajji et al., 2022). Fluorescence intensity given in MESF units can be then compared to other flow cytometers.

#### 2.2.4 Novel instruments

Notably, there are some custom-made nFC instruments that enable the detection of nanoscale particles; however, the repeatability between instruments constructed in this manner is unknown. There are also newly developed instruments dedicated to small-particle analysis using nFC that may provide valuable information about EV molecular cargo after proper validation. All these instruments are listed in Table 1. However, their performance requires time to be comprehensively evaluated and compared to other instruments, along with the establishment and evaluation of consistent labeling protocols (Lopez-Pacheco et al., 2021; Salmond et al., 2021).

##### 2.2.4.1 Imaging flow cytometry

Another type of FC that provides higher fluorescence sensitivity and resolution than traditional FC and can be used for EV analysis is imaging flow cytometry (IFC). The main change in conventional FC is the way in which the optical signal is detected and processed. These cytometers use charge-coupled device (CCD) cameras, which have lower noise and broader dynamic range than PMTs from conventional cytometers. Additionally, IFC instruments have a so-called time delay integration (TDI) of pixel intensities on CCD cameras and slower flow rates; therefore, the signal has longer integration for each particle, which increases the sensitivity. Moreover, the images of all events in all channels are stored so that they can be processed for further data analysis (Botha et al., 2021). Still, most EVs are below the diffraction limit and are visualized as diffraction-limited spots.

IFC encounters several technical issues related to signal processing, that cause the necessity for appropriate calibration, gating strategy, controls and serial dilutions (Woud et al., 2022; Welsh et al., 2023; Wu et al., 2023). Interestingly, in case of IFC, staining intensity and sample recovery vary depending on the temperature of the incubation with fluorescent antibodies (Tertel et al., 2020). To prevent coincident detection in imaging FC, Woud et al. proposed a specific gating strategy, were they collected events displaying 0 or 1 fluorescent spot on acquired images. This ensured that they analyzed only single particles and not multiple particles. The authors also suggested standardizing SSC signal intensities for the estimation of particle sizes and colocalization of at least two fluorophores to assess the presence of two markers on the same particle. They underlined the importance of a detergent-treated sample as a control; the disappearance of the signal after detergent treatment ensures that the detected fluorescent events are associated with lipid membranes of biological origin (Woud et al., 2022).

##### 2.2.4.2 Spectral flow cytometry

An additional type of FC for EV characterization is spectral flow cytometry (SFC) (Aibaidula et al., 2023). In which optical signals are collected from the full emission spectrum, not only from the section where each fluorochrome has an emission peak. Collecting the entire spectrum reveals differences in the pattern between fluorophores with similar emission peaks and allows more fluorochromes to be used to stain a single sample. These spectrometers apply a spectral unmixing procedure to unravel the signal from each fluorophore. However, the use of SFC for EV analysis is limited by the small surface area and dim signals of EVs similar to nFC (Welsh et al., 2023).

## 3 Labeling

### 3.1 Sample handling: impact of EV isolation method, sample concentration and background

EV labeling protocols are often based on protocols and dyes that were initially dedicated to cells. However, owing to the several orders of magnitude smaller size of EVs, these protocols must be adapted to meet the special requirements and challenges connected to EVs, which are discussed in more detail later in this chapter.

In general, it is important to optimize dye concentration, staining temperature, and EV purification methods before conducting actual sample measurements. Midekessa et al. showed, for example, that the size of fluorescent particles decreases and their number increases with higher concentrations of the lipophilic dye Cell Mask Green (CMG) (Midekessa et al., 2021). Those differences may be related to the differences in apparatus sensitivity in the scatter and fluorescent modes. Because of the low refractive indices of sEVs, they may not reflect enough light to be detected in the scatter mode; however, after labeling, the fluorescent signal is much more intense, and these particles can be detected in the fluorescent mode. Because, as mentioned above, particular impurities affect fluorescent staining and subsequent f-NTA measurements, the chosen EV isolation method will influence the subsequent staining and f-NTA results. Midekessa et al. observed that the number of CMG-stained EVs increased slightly with the incubation temperature. This can be explained by the higher fluidity of the double phospholipid bilayer of EVs at higher temperatures, which favors intercalation of dye molecules into the membrane. The EV purification method impacted their NTA results - in the case of combination of tangential flow filtration (TFF) and SEC the authors detected fewer particles but with a bigger size than using only the SEC method (Midekessa et al., 2021). The authors explained that these results showed the impact of the purification method used for EV preparation on f-NTA measurements–by combining TFF and SEC they obtained a different particle composition in the analyzed sample, which was reflected by the EV profile detected by NTA.

Interestingly, Koksal et al. mentioned that every precipitation and centrifugation step during EV preparation for analysis due to mechanical stress influences the EV conformation and activity of surface markers. Consequently, fewer EVs can be detected using fluorescent antibody labeling methods (Koksal et al., 2023). The authors admitted that f-NTA is a time-consuming and operator-specific method. The duration of the entire analysis must be within the range of fluorochrome optimal glowing properties to prevent the impact of photobleaching. The samples were protected from light during the entire protocol for all the washing and measurement steps. Altogether, the difficulties described above limit the applicability of f-NTA as an EV analytical method, particularly in the clinical context.

Another important factor affecting NTA results is the sample concentration. Although the sample dilution factor is considered by the analysis software for concentration calculations, one must be within the optimal concentration range of the sample for the measurement. Sałaga-Zalewska et al. noticed that too high or too low sample dilution during measurement distorts the determination of the total number of particles per milliliter (Salaga-Zaleska et al., 2023). Too many particles in the field of view during NTA measurement can lead to particle interaction, collision, and overlapping, which may interfere with particle movement and give unreliable results (Yahata et al., 2021). It is also a known effect in flow cytometry measurements of EVs and is recognized as the swarming effect. Therefore, the sample concentration for the measurement must be carefully optimized. Furthermore, in the case of f-NTA, the optimal concentrations for the scatter and fluorescent modes may be different. In most cases, we aim to determine the absolute number or percentage of our fluorescent-positive particle (EV) population relative to the total particles, and a measurement in both modes (scatter and fluorescent) of exactly the same sample loaded into the flow cell is needed. The concentration of the sample optimal for scatter light analysis may be too low for measurement in fluorescent mode, especially if the percentage of the fluorescent-positive EV population is low, for example, the number of tetraspanin CD9 positive EVs–Thus, we have too few fluorescent particles in the field of view for accurate concentration calculations by the software. In contrast, when we adjust the sample concentration to be optimal for fluorescence measurement, it might be too high for the scatter mode, where many more particles will be detected by the instrument, and there is a risk of the swarming effect. Furthermore, the optimal sample dilution for an f-NTA measurement also depends on the background of the dye or fluorescent antibody and sample impurities.

Often, as previously mentioned, other compounds are co-isolated with EVs, such as large protein complexes, soluble proteins, and cell culture media components. These compounds can significantly influence the effectiveness of the labeling and results. Their influence is discussed in the third section of this review. There should always be a negative (the sample containing the solvent without EVs, but treated and labeled the same way as EV sample) and a background control (the sample containing only the solvent or the sample matrix) to provide reliable results (Fortunato et al., 2021). In addition, depending on the dye or type of fluorescent antibody, staining may also give a more or less high background signal in the fluorescent mode due to the formation of micelles or aggregates detectable by the instrument, unspecific binding to sample contaminants, or other undefined reasons. This background influence can be reduced by a high dilution of the sample after staining for measurement on the NTA instrument. However, the requirement for this is an initially highly concentrated EV sample for the staining step, which ensures that after the high dilution for measurement (at least 100 times), the EV number in the field of view remains sufficiently high to be in the range required for the measurement. Therefore, it is necessary to balance the initial EV sample concentration for staining, dye or antibody concentration, and dilution for measurement to obtain an optimal result, which has been shown in our study (Dlugolecka et al., 2021). Alternatively, a washing step can be performed after staining to remove unbound dye or antibody (discussed in detail below); however, this step is not always applicable and can contribute to the loss of EV samples.

### 3.2 EV labeling efficiency, nonspecific labeling

EV labeling techniques used for fluorescence analysis by f-NTA, nFC, or other methods present many challenges. One of the important issues is labeling efficiency. During analysis, the concentration of total particles in scatter (on NTA) and labeled particles (f-NTA) can be compared, but the actual efficiency of labeling “real” EVs is unknown (Dlugolecka et al., 2021). The total particle concentration, in fact, counts particles that are EVs and are labeled, particles that are EVs but because of labeling efficiency will not be labeled and particles that are not EVs. Researchers must be aware of this during the data analysis.

Interestingly, Chen et al., using nFC, noticed a large variation in the labeling performance of lipophilic dyes or lipophilic membrane probes, probably because of the heterogeneous nature of EVs and the differences in their lipid composition. Their results showed that the labeling efficiency of EVs differed according to different biological sources, such as different cell lines, and varied within individuals for EVs from plasma (Chen et al., 2023).

Similar observations were made by Tertel et al., who compared the efficiency and specificity of common EV dyes. They stained MSC-derived EVs with a few conventionally used dyes (BODIPY TR ceramide, calcein AM, CFDA-SE, PKH67, and Exoria) and analyzed them by Imaging FC. Additionally, to determine specificity, labeled objects were treated with detergent NP-40. Only events that disappeared after detergent treatment were considered true EVs. The objects labeled with CCFDA-SE and BODIpY TR ceramide were not affected by detergent treatment; therefore, the authors concluded that those were not small EVs. Calcein AM failed to stain any object. Only PKH67 and Exoria dyes successfully stained EVs based on light-scattering properties and detergent control. Co-staining using fluorescently labeled antibodies against tetraspanins showed that, in the case of MSC-derived EVs Exoria was more specific to tetraspanin-positive particles than PKH67. The authors also mentioned that the labeling results differed depending on the source of EVs and their molecular content. For instance, different cell types secrete EVs with varying esterase contents, which limits the utility of CFSE as a dye for cell types with low intracellular and intra-vesicular esterase concentrations (Tertel et al., 2022).

Notably, Melling et al. performed an interesting study in which they labeled EVs previously tagged with mEmerald-CD81 with two types of dyes, PKH26 and C5-maleimide-Alexa633. They performed colocalization tests for both the dyes and CD81 using confocal microscopy. The results showed that most of the tagged EVs were not labeled with either PKH26 (only 4.6% ± 1.6 was labeled), or C5-maleimide-Alexa63 (35.4% ± 1.8 was labeled) (Melling et al., 2022). The authors noticed that a significant fraction of the dye was not associated with EVs. They observed additional particles by NTA and confocal microscopy in the dye controls, which corresponded to macromolecular dye self-aggregates and micelles. Notably, after an additional cleaning step using SEC, the authors noticed the elimination of this maleimide signal and the reduction of the PKH26 signal in the dye controls. A substantial amount of signal from both dyes was detected after an analogous cleaning step with an Exospin column. The authors suggested that EV-staining dyes can form large molecular aggregates, but certain techniques can be employed to minimize their occurrence (Melling et al., 2022).

The formation of micelles and aggregates in the PKH26 dye was also reported by Morales-Kastresana et al., 2017. The researchers observed a higher event rate in the EV sample labeled with PKH26 on nFC and NTA than in the unstained EV sample. A non-EV control (PBS + PKH dye) also showed a differentiated particle distribution, corresponding to the presence of micelles or PKH26 aggregates (Morales-Kastresana et al., 2017). Furthermore, the authors also assessed CFSE as an EV dye and observed that there was no evidence of micelle or aggregate formation, since the concentration and size distribution of the labeled sample remained similar to that of the unstained EVs. However, there was a shift in the fluorescence of the background reference noise events on the nFC, which corresponded to the unbound dye. To reduce background fluorescence, the authors used several techniques (SEC, UCF, sucrose cushions, or CFSE sequestration with BSA-coated beads) and reported that SEC was the most effective in removing unbound labels (Morales-Kastresana et al., 2017).

Additionally, Fortunato et al. emphasizes that, for example, CFSE can give an unspecific signal from the contamination with soluble esterases (Fortunato et al., 2021). Loconte et al. analyzed EVs labeled with several dyes: MG-488, CFDA-SE, or labeled through the expression of a mp-sfGFP and evaluated uptake experiments by spectral flow cytometry and imaging flow cytometry. They found that EVs labeled with MG-488 were present in all cell types, EVs stained with CFSE were only visible in a minor subset of cells, and EVs labeled with mp-sfGFP were mostly detected in CD14^+^ monocytes. The authors stated that all combined methods provided complementary information about EVs (Loconte et al., 2023).

Some studies have described possible solutions to increase the efficiency of lipophilic dyes. Cha et al. proposed reducing the NaCl concentration of the buffer during labeling to 20 mM NaCl to help lipophilic dyes enter the membrane (Cha et al., 2023). They explain that lipophilic dyes are not getting efficiently incorporated into vesicle membranes in an aqueous buffer because of their low water solubility. At lower salt concentrations, the dye was more dispersed and better available for vesicle membrane incorporation. After labeling, they suggested increasing the ionic strength to 150 mM NaCl because the dye forms macromolecular aggregates that can be easily separated from vesicles by regular syringe filtration using 0.2 µm filters. A comparison between conventional staining and salt-change staining showed a much higher efficiency of the salt-change method. It has been shown to work with several types of vesicles and lipophilic dyes, such as DiI, DiD and PKH67 (Cha et al., 2023). Moreover, their experiments showed that, using the salt change method, less dye is needed for satisfactory results, and because there is a small amount of dye molecules per vesicle, the impact of the dye on vesicle characteristics such as size and functionality is minimal.

In antibody labeling, the selection of a specific type and clone is critical, as their performance can vary depending on the assay type and conditions. To assist in the selection of the appropriate antibody and to minimize the need for extensive optimization studies, the EV Antibody Database has been established (Morey et al., 2024). Although currently limited, the database is open access and is intended to provide detailed information on assay variables and protocols in the future, to support the sharing of relevant antibody data in EV research. It includes information on antibodies tested in Western blots, flow cytometry, and other assays, helping researchers eliminate inefficient antibodies from their protocols and select more effective ones. Also, the proper antibody to sample ratio during the staining process is critical to results, and this information can also be included in the database.

According to staining with fluorescent antibodies directed against certain EV surface markers, the observed variability in staining efficiency is an effect not only on the staining performance of a given antibody, but also on the heterogeneous marker expression of EV populations. Interestingly, Spitzberg et al. performed a multiplexed analysis of EVs using high-resolution microscopy (MASEV), the method of direct stochastic optical reconstruction (dSTORM), and self-made microfluidic devices (Spitzberg et al., 2023). In which the authors investigated whether common EV markers used in bulk methods, such as Western blotting and ELISA, are present in variable concentrations in all EVs or if some EVs are enriched in specific proteins. Their analysis revealed that there is in fact a heterogeneous distribution of specific markers across all EV groups. The most abundant protein was CD9 (47.9%) in the PANC-1 cell line. They also evaluated the concomitance of the different biomarkers in each vesicle. The results revealed that many of the tested EVs had a low percentage or no tetraspanins depending on the cell line. This implies that in the case of affinity purification of EVs using one of the tetraspanin markers from biological fluids, an unknown, but in some cases, a substantial number of EVs could be missed. The authors suggested that it is worth to use pan-tetraspanin affinity purification to raise the ratio of isolated vesicles and increase the detection yield (Spitzberg et al., 2023). Other studies also show that tetraspanins are not expressed evenly across different EV sources and that the tetraspanin profile changes depending on EV size, subclass and source (Mizenko et al., 2021).

These studies indicate that none of the methods currently used for labeling EVs offers accurate quantitative measurements. Rather, samples can only be compared among themselves, as the number of stained EVs is often overestimated owing to dye and background aggregates. In addition, it is difficult to assess staining efficiency, which affects the precision and reliability of the obtained results.

### 3.3 Does the EV staining method impact the functionality of EVs?

Loconte et al., in their work mentioned in the previous chapter, reported that the labeling of EVs considerably influences their interactions with recipient cells, including their uptake and cargo delivery. EVs labeled with MG-488 were found in all cell types, and the same EVs labeled with other dyes were detected in only some cell subsets. This indicated that the labeling type considerably affected EV functionality in the uptake experiments. Therefore, the authors concluded that combined labeling methods could provide more complete information about the interaction of EVs with cells (Loconte et al., 2023).

Furthermore, Chen et al. performed a functionality test of EVs labeled with the lipophilic membrane probe DSPE-PEG_2000_-biotin to check if there was a steric hindrance effect impacting surface protein analysis during labeling with PE-conjugated antibodies against CD9, CD63, and CD81. No impact of this lipophilic membrane probe on the antibody staining or functionality of EVs was observed (Chen et al., 2023).

In their review of labeling techniques, Bao et al. highlighted the advantages of using aptamer particles instead of classical antibodies. Conventional antibodies can induce immunological reactions and EV aggregation, which can affect EV properties and functionality *in vivo*. Aptamers are short stretches of nucleic acids (DNA or RNA) or peptides that can bind specifically to specific molecules, such as proteins, small organic molecules, metal ions, and even whole cells. Aptamers act similarly to antibodies, showing high specificity and affinity for their targets but differ in structure and production methods. Compared to classical fluorescent antibodies, aptamers are much smaller, have higher biocompatibility, and do not affect the physicochemical and biological functions of EVs. In addition, aptamers can be chemically synthesized, allowing precise control of their sequences and properties. The aptamer selection process can be performed completely *in vitro*, whereas antibodies are typically produced in living organisms (Bao et al., 2023).

Moreover, Arifin et al. described current state-of-the-art imaging techniques for studying EV uptake and distribution *in vivo,* focusing on the biodistribution and pharmacokinetic profiles of EVs after administration *in vivo.* The authors reported cytotoxicity at higher label concentrations, which may severely impact EV and cell functionality; therefore, optimizing the label concentration is important to lessen the cytotoxic effect *in vivo*. Additionally, the authors observed an altered surface charge or size distribution of EVs at high label concentrations, which may also influence EV functionality (Arifin et al., 2022).

The most common labels used in EV studies are listed in Table 2, which also addresses their advantages, disadvantages, and impact on EV function.

**TABLE 2:** Common label types.

Name of the label	company	type	advantages	disadvantages	impact of staining on EV function	application	References
CellMask	Thermo Fisher Scientific	lipophilic membrane dye	membrane-specific labeling, compatible with live-cell imaging, slow internalization, can be used after fixation	label every biological compound with a lipid membrane, not only EVs, cannot be used after permeabilization, detergent sensitive, impacts the size of EVs	may affect EV uptake and cargo distribution	EV membrane labeling	Dlugolecka et al. (2021), Midekessa et al. (2021), Bao et al. (2023)
Specific antibody conjugate with fluorescent probe like CD9-PE, CD9-AF488, etc.	Various	immunospecific, protein-specific	highly specific labeling of target proteins	potential for nonspecific binding, binding many proteins causes increase of EVs size, for most applications only labeling of surface markers, fluorophore size may cause steric hindrance	may influence EV protein function and sorting, obscure functional receptors on EV surface, affects EV physiochemical properties and biological functions	EV protein labeling	Dlugolecka et al. (2021), Bao et al. (2023)
CFDA-SE (CFSE)	Thermo Fisher Scientific	amine-reactive become fluorescent after enzymatic reactions	stable and covalent labeling of cellular components, allows to study EV internalization and content transfer *in vitro*, allows the detection of intact EVs and their content delivery	limited to intact EV labeling, EVs from different sources can differ in esterase content and therefore its staining efficiency depends strongly on source of EVs	may alter EV uptake and cargo distribution; do not perturb the size of EVs nor their biodistribution	EV labeling, tracking	Morales-Kastresana et al. (2017), Dehghani et al. (2020), Barrachina et al. (2022), Tertel et al. (2022), Loconte et al. (2023)
PKH67	Sigma-Aldrich	lipophilic dye	bright fluorescence, compatible with various imaging modalities	limited to lipid membrane labeling, creates aggregates and micelles, so washing step is necessary	may affect EV membrane properties, impacts EV size	EV labeling, imaging *in vivo* and *in vitro*	Liebel et al. (2020), Droste et al. (2021), Bao et al. (2023), Cha et al. (2023), Chen et al. (2023)
PKH26	Sigma-Aldrich	lipophilic dye	high stability, long-lasting fluorescence	requires optimization for different EV types, creates aggregates and micelles, so washing step is necessary	impacts EV size	EV tracking, imaging *in vivo* and *in vitro*, functional studies	Morales-Kastresana et al. (2017), Puzar Dominkus et al. (2018), Dehghani et al. (2020), Melling et al. (2022), Bao et al. (2023), Chen et al. (2023)
DiI, DiO, DiL, DiD, DiR	Thermo Fisher Scientific	lipophilic dye	bright fluorescence, long-term labeling, minimal background fluorescence, stable staining, almost no staining transfer between EVs	photobleaching over time, creates aggregates and micelles	may alter EV uptake and cellular response	EV labeling, imaging *in vitro*; DiD, DiR *in vivo* imaging	Rautaniemi et al. (2021), Bao et al. (2023), Cha et al. (2023), Chen et al. (2023)
Azido-dPEG-TFP ester, linked inhouse with AF350, AF488, or AF647	Quanta Bio-design	fluorescently label free amines of EV-surface proteins	labels any accessible EV surface protein equally well, bright, stable, PEG linker increases water solubility, labeling efficiency and reduce nonspecific EV binding/aggregation	increases size of EV, impairs function of EV surface proteins	may influence EV biodistribution and interactions with target cells	total EV labeling	Ferguson et al. (2022), Spitzberg et al. (2023)
C5-maleimide-Alexa633	Thermo Fisher Scientific	thiol-reactive	strong fluorescence, selective labeling	potential for nonspecific binding	may influence EV stability and uptake	EV labeling, imaging	Roberts-Dalton et al. (2017), Melling et al. (2022)
MemBright	BioActs	membrane-specific	high specificity, compatible with live-cell imaging, simple handling, great specificity, low working concentration, no cytotoxicity, compatible with many fluorescence imaging techniques, no aggregates	limited to membrane labeling	may alter EV membrane properties	EV membrane labeling	Collot et al. (2019), Melling et al. (2022), Bao et al. (2023), Boudna et al. (2024)
MemGlow (MG)	BioActs	membrane-specific	bright fluorescence, minimal background; creates non-fluorescent aggregates, allows the detection of short/transient interactions	potential photobleaching, possible dye transfer through brief interaction with the recipient cell	may affect EV uptake and cargo distribution	EV membrane labeling	Loconte et al. (2023), Rodriguez et al. (2023)
ExoGlow	System Biosciences	membrane-specific	bright, intact membrane specific	can also label liposomes and lipoproteins	may alter EV membrane properties	EV membrane labeling	Kamei et al. (2021), Roseborough et al. (2023)
ExoTracker	SBI	fluorescent	compatible with EV tracking in live cells	limited to fluorescence microscopy	may alter EV distribution and cargo sorting	EV tracking	Zhou et al. (2020), Loconte et al. (2023)
DHPE	Sigma-Aldrich	lipophilic dye	stable incorporation into lipid bilayers	limited compatibility with certain imaging modalities	may influence EV membrane properties	EV labeling, membrane studies	Nazarenko et al. (2013), Rautaniemi et al. (2021)
Ptx-OG (paclitaxel Oregon Green)	Creative Bioarray	fluorescent dye conjugate of the chemotherapy drug paclitaxel	selective labeling, minimal interference, high fluorescence quantum yield, photostability	potential for nonspecific binding, paclitaxel cytotoxicity	may impact EV protein function and sorting	EV uptake and intracellular trafficking	Saari et al. (2018), Rautaniemi et al. (2021)
SYTO RNASelect	Thermo Fisher Scientific	nucleic acid-specific	highly specific for RNA, compatible with flow cytometry	limited signal intensity in EVs with low RNA content	minimal impact on EV function	analysis of EV RNA cargo	Popena et al. (2018), Adamo et al. (2019), Melling et al. (2022)
Exoria	AAT Bioquest	lipophilic dye	high photostability, compatible with flow cytometry	limited spectral range, potential cytotoxicity	may affect EV uptake and cargo distribution	EV labeling, tracking	Chong et al. (2022), Tertel et al. (2022), Johnson et al. (2023)
Calcein AM	Abcam	fluorescent dye	non-toxic, suitable for live-cell imaging	limited membrane permeability	minimal impact on EV function	monitoring EV release dynamics	Gray et al. (2015), Tertel et al. (2022)
BODIPY and derivatives (Dp ceramide, BPC12, BP and others)	AAT Bioquest, Creative Bioarray	lipophilic dye	bright fluorescence, high quantum yield, long-term labeling, sharp absorption, and emission peaks, and good photostability, easy to modify to adjust photophysical properties, biocompatible	possible unspecific binding, time consuming modification procedure, limited effectiveness in deep tissue imaging	may alter EV membrane properties	uptake and trafficking inside the cell	Rautaniemi et al. (2021), Tertel et al. (2022)
CellTracker Red CMTPX	Invitrogen	fluorescent dye	highly stable, compatible with live-cell imaging	moderate photostability, pH-dependent fluorescence	minimal impact on EV function	studying EV uptake and trafficking	Tong et al. (2017), Reginald-Opara et al. (2022), Song et al. (2023)
Di-8-ANEPPS	Invitrogen	lipophilic dye	high sensitivity to membrane potential changes, Suitable for membrane potential imaging in live cells, high effectiveness of EV labeling	toxic at high concentrations, Limited selectivity for specific membranes, creates aggregates and micelles	potential disruption of EV membrane integrity	membrane potential imaging, live-cell studies	Chen et al. (2023), von Lersner et al. (2024)
DSPE-PEG_2000_-biotin	Avanti Polar Lipids, Inc	lipophilic membrane probe	biotinylated for specific interaction with streptavidin or avidin, PEG linker enhances solubility and stability	potential alteration of lipid bilayer properties, requires streptavidin or avidin for detection	minimal impact on EV function in case of interaction with antibodies for tetraspanins	biotinylation of EVs for isolation or detection	Wan et al. (2017), Chen et al. (2023)
GFP	-	fluorescent protein	adequate to follow the first steps of uptake, bright green fluorescence	requires genetic engineering for expression, may interfere with protein function if fused improperly	may interfere with protein function if fused improperly	visualization and tracking of proteins, organelles, cellular structures (including EVs) in living cells	Corso et al. (2019), Loconte et al. (2023)

### 3.4 Washing after labeling

As presented above, a substantial amount of background and unspecific staining can be expected under certain circumstances during fluorescent labeling of EVs, and a washing step is highly recommended. There are a few ways to perform washing after labeling, like SEC, UCF, ultracentrifugation with a discontinuous density gradient (UCG), ultrafiltration (UF), anion exchange chromatography (AEC) or with affinity beads (Morales-Kastresana et al., 2017; Fortunato et al., 2021; Rautaniemi et al., 2021). Unfortunately, washing always causes some loses of the stained material, which is especially problematic in the case of small amount of original sample material (for instance, from biological fluids), and also due to this losses the following quantitative measurement is biased. Moreover, the efficiency of the removal of unbound dyes is strongly dependent on dye properties. Rautniemi et al. concludes that for a good purification the relative purification efficiency (E_rp_; recovery of the EVs divided by the recovery of the dye) should be higher than one (Rautaniemi et al., 2021). The best method of EV purification after labeling found in their work was SEC. However, after purification, stained EVs need to have a sufficiently high fluorescence intensity to be visible in the target application, and in their case, the fluorescence of labeled and purified EVs was too weak to be detected after administration to cells.

In some cases, when the amount of biological sample is very limited, dilution can be performed instead of washing to reduce the background from the unbound dye as much as possible. This is often done in the case of NTA, where the sample has to be strongly diluted to be within the detection range. Detergent lysis controls, buffer controls without EVs, and unstained samples for antibody labeling must be provided even after washing to control for background signal and unspecific staining (Gorgens et al., 2019; Dlugolecka et al., 2021; von Lersner et al., 2024).

## 4 Lipoproteins and corona

**FIGURE 2:**
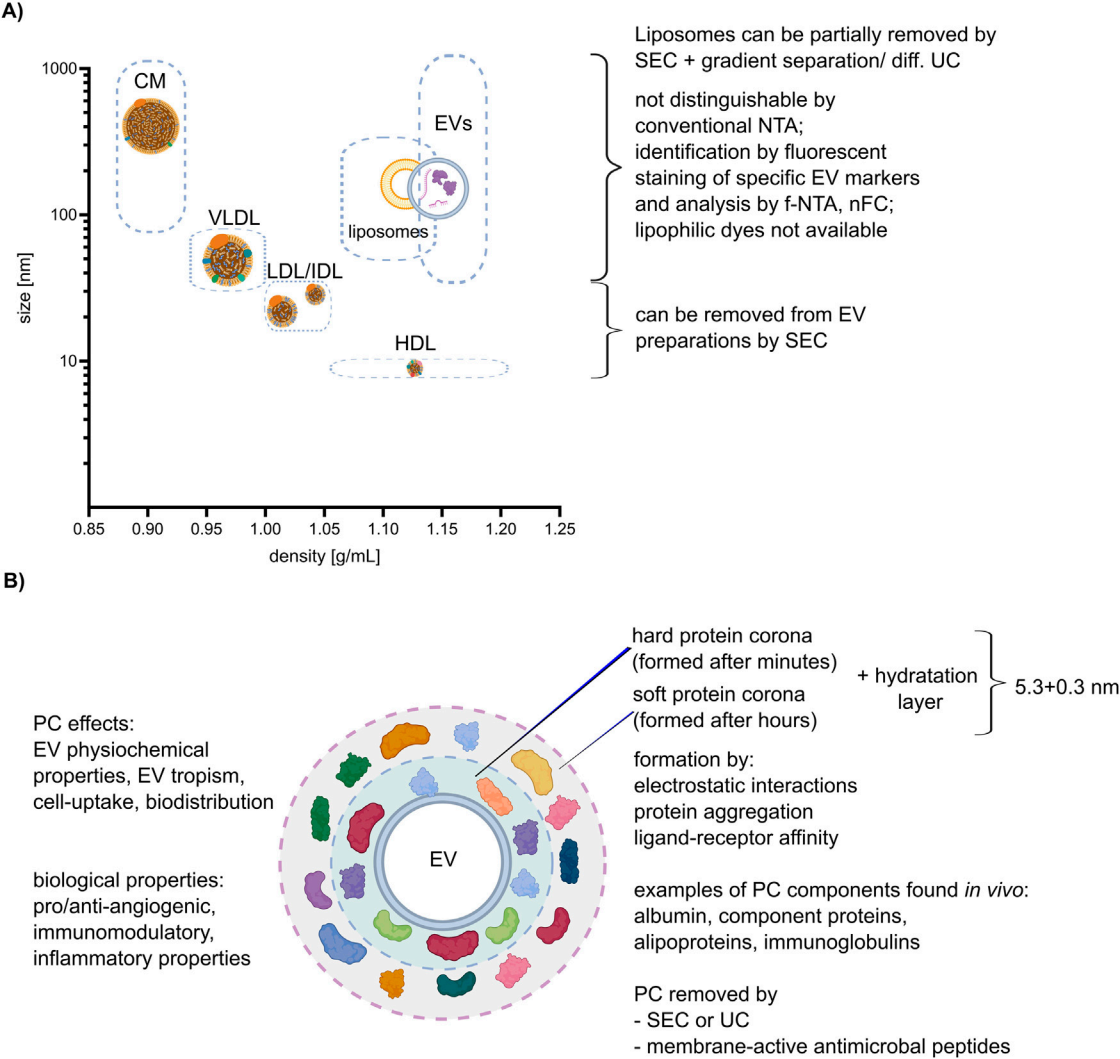
Lipoproteins and corona. **(A)** Correlation between size and density of lipoproteins, liposomes, and EVs (size and density ranges are depicted by dashed boxes), with an explanation of possible separation methods. The different lipoprotein classes: chylomicron (CM), high-density lipoprotein (HDL), low-density lipoprotein (LDL), very low-density lipoprotein (VLDL). **(B)** Characteristics of the EV protein corona (PC) and its impact on EV properties and function. Created with BioRender.com and Inkscape.

Lipoproteins are biochemical complexes of lipids such as triglycerides and phospholipids, with special proteins called apolipoproteins. Their primary function is to transport hydrophobic lipids (also known as fat) in the blood plasma or other extracellular fluids. Plasma lipoproteins are typically divided into five main groups based on their size, lipid composition, and apolipoprotein content, which are very low-density lipoproteins (VLDLs), intermediate- and low-density lipoproteins (IDLs and LDLs), high-density lipoproteins (HDLs), and chylomicrons (Simonsen, 2017). They outnumber plasma EVs by orders of magnitude and can be co-isolated with EVs during the separation process, leading to potential contamination or interference with the EV staining process as shown on Figure 2A (Chen et al., 2023; Lozano-Andres et al., 2023; Boudna et al., 2024). In conventional light scattering, NTA EVs cannot be distinguished from lipoproteins of similar size. Labeling with lipophilic dyes, as shown recently, will unfortunately not help to distinguish EVs from lipoproteins, since both EVs and lipoproteins are labeled due to their phospholipid membrane. Therefore, lipoproteins affect the accuracy and specificity of labeling (Chen et al., 2023). The most frequently used method for plasma EV separation is SEC (Pang et al., 2020). It enables the purification of EVs from LDLs and HDLs because of their difference in size, but not from VLDLs and chylomicrons. A combination of SEC and additional gradient separation or differential UC can additionally remove more lipoproteins, although it also lowers the total particle count (Karimi et al., 2018). Notably, in case of plasma sample collection it was proven that, regardless of the chosen EV purification method, it is advisory to collect blood samples in pre-prandial state to reduce lipoproteins contamination (Tushuizen et al., 2012).

The protein corona (PC) is the areola of biomolecules, including proteins and lipids, which form around EVs when they come into contact with biological fluids as shown on Figure 2B (Toth et al., 2021). Those molecules are attached to EVs not covalently but by other interactions, such as hydrogen bonds, van der Waals interactions, and electrostatic interactions. Varga et al. shows that the thickness of the hydration layer (including PC) can be calculated by combining optical methods like dynamic light scattering (DLS) and NTA, and non-optical methods like microfluidic resistive pulse sensing (MRPS) and very small-angle neutron scattering (VSANS) and is around (5.3 ± 0.3) nm (Varga et al., 2020).

The PC can alter the surface properties and size of EVs and impact their interactions with recipient cells (Varga et al., 2020). During the labeling process, the PC may affect the accessibility of the labeling agent to the surface of EV, potentially reducing labeling efficiency or specificity. In addition, some proteins or lipids present in the PC can be labeled, but they are not a physical part of the EV themselves. With standard staining, we cannot distinguish between what is a real EVs surface marker and what is only a component of the corona, and it can be distinguished only after removing it. Yahata et al. claimed that the observed differences between liposome and EVs properties potentially originated from the PC (Yahata et al., 2021).

To remove the PC Singh et al. proposed the use of membrane-active antimicrobial peptides (AMPs). Those AMPs typically have a short sequence (10–50 residues), mostly a net positive charge, and contain ∼ 50% hydrophobic residues that make them membrane active (Mangoni et al., 2015; Nayab et al., 2022). They can approach the surface of a lipid bilayer in such a way that associated proteins can be removed from EV surfaces. Comparison of control EVs with AMP-treated samples revealed detachment of proteins adsorbed on the lipid bilayer of EVs (Singh et al., 2020; Singh et al., 2023).

Interestingly, Wolf et al. demonstrated the significant influence of PC removal (by a subsequent process of EV separation using TFF followed by SEC or UCF) on angiogenesis and immunomodulation. Their results showed that these functions are closely linked to the presence of the PC, and once it is removed, these functions are lost (Wolf et al., 2022). Additionally, Toth et al. showed that the labeling results differed greatly depending on the composition of the PC. They incubated medium-sized EVs (100–800 nm in diameter, typically sedimented at 10,000–20,000 × g) isolated from THP-1 cells with EV-depleted blood plasma from patients and then characterized the coated EVs using several methods. Nascent EVs, plasma protein aggregates, nascent EVs incubated with fibrinogen, and annexin V-positive plasma EVs were used as the controls. In addition, the authors demonstrated that EVs with an external plasma protein cargo, in contrast to nascent EVs, induced increased expression of TNF-α, IL-6, CD83, CD86, and HLA-DR in human monocyte-derived dendritic cells (Toth et al., 2021).

Notably, an increasing number of studies suggest that PC and lipoproteins should not always be perceived as contamination but may play an important role in the biological function of EVs, and functional studies should carefully investigate whether isolating EVs from PC is the best solution (Dietz et al., 2023; Liam-Or et al., 2024; Welsh et al., 2024). When researchers plan labeling approaches for their isolated EV samples for functional testing, they need to consider the impact of PC and lipoproteins on their results.

## 5 Development of EV labeling towards medical applications - challenges

The number of studies using fluorescent labeling of EV for the development of future clinical diagnostic or prognostic applications is increasing exponentially every year. However, many published studies present controversial results or outcomes that are difficult to assess, interpret, and compare with other results. The reason for this is often an incomplete description of the methods and results in manuscripts, an inappropriate experimental design, lack of appropriate calibration and standardization and mistakes in interpreting results–like, for example, considering actual measurement artifacts as “true” EVs. A few years ago, EV researchers proposed a flow-cytometry-specific reporting framework of EV studies that included detailed guidelines regarding methods and data reporting, which will allow a full interpretation and validation of flow cytometry data of EVs (Welsh et al., 2020). These guidelines, although initially referring only to flow cytometry, it can be easily applied to all other EV analysis methods that are based on fluorescent staining. A broad implementation of this reporting framework in experimental practice is necessary for the development of standardized, reliable, and validated fluorescence-based EV analysis methods that can be implemented as clinical diagnostic or prognostic tools in the future.

Although an increasing number of researchers have attempted to follow in their manuscripts the guidelines mentioned above, there are still studies that lack important controls or information about critical variables. In many of these studies, the authors failed to provide all the necessary information for evaluation if all relevant disruptive factors for staining have been appropriately reviewed and assessed. Based on selected examples of reported studies, we briefly discuss the common mistakes and misinterpretations within fluorescent-staining-based EV experiments and propose possible improvements.

In his study, Koksal et al. presented a quantitation of cancer-derived EVs in clinical samples of hepatocellular carcinoma (HCC), based on fluorescent staining of typical HCC markers and subsequent f-NTA analysis (Koksal et al., 2023). The authors showed that by this method they can discriminate between HCC patients and cirrhosis patients, and that the presented EV quantification correlated with the size of the liver tumor assessed by liver imaging. The authors have correctly reported the NTA instrument settings and staining procedures in detail. However, they did not avoid mistakes in experimental design or reporting. The optimization of antibody and dye concentrations was performed only on EVs isolated from the liver cancer cell line Huh7, even though the assay was performed directly in the serum. Admittedly, the authors mentioned in the Methods section that they had also tested several antibody dilutions in serum samples, but they did not show or report any results. It is expected that in the tested serum samples, there will be many proteins and impurities that will impact fluorescent staining and may lead to a much higher number of staining artifacts in comparison to the isolated EV samples from cell culture used for staining optimization. The much higher percentage of membrane-stained particles observed in the serum samples compared to the cell-culture EV-samples implies not necessarily, as interpreted by the authors, a higher EV content, but may be also a sign of unspecific staining due to the serum background. Staining and analysis of appropriate controls, consisting of Huh7-derived EVs spiked into serum samples or EV-free serum samples, would help to evaluate the impact of background staining. The signal linearity and concentration dependence of NTA measurements with serially diluted serum samples are not presented. Additionally, antibody and dye concentrations were reported only as relative dilutions and not in appropriate absolute units, such as mole/L or mg/mL. Furthermore, it was not stated if any antibody/dye-only controls were used to assess background dye staining. It is not clear how the SEC washing step was optimized, especially how EV recovery was evaluated against EV quality performance. The actual fractions that were collected and analyzed after sample application on the given column were not stated. It is not clear why the recovery and quality assessment of the samples after the column wash were analyzed only in scatter mode, which does not provide any information regarding the number of stained EVs. A high particle number in this case may indicate a high impurity content and pure column performance, rather than a high EV recovery. The pre-analytical variables according to serum collection were reported very superficially and did not provide details about the blood collection and centrifugation steps to obtain the serum.

In another study, the authors perform labeling only with the CMG dye and did not check for specificity, staining saturation, or background staining (Piibor et al., 2023). The significantly higher particle concentration of the “Total-NP” fraction after CMG-staining compared to the “EV only” fraction of unlabeled particles measured in the scatter mode may imply the formation of some kind of aggregates (e.g., of CMG) during staining. The possible impact of their presence on NTA measurements in fluorescent mode was not evaluated (e.g., by the measurement of a dye-only sample at the same concentration).

Table 3 presents a summarized list of factors that should be considered in the fluorescence analysis of EVs in selected studies, including those discussed above. Researchers must be cautious when attempting to draw clinical conclusions based on limited information. None of these studies have examined the impact of labeling on EV functionality, which can have a substantial meaning for clinical outcomes. This table shows that there is still a long road to make solid, evidence-based conclusions from EV-based studies. Substantial dose improvements and additional experiments are required to provide reliable data.

**TABLE 3:** List of factors that should be considered in the fluorescent analysis of EVs.

Article	Koksal et al. (2023)	Piibor et al. (2023)	Bhagwan Valjee et al. (2024)
Method type	F-NTA	F-NTA	Plasmon resonance
Device name for fluorescence detection	Nanosight, Malvern Panalytical	ZetaView, Particle Metrix	ExoView, NanoView Biosciences
Labeling type	immunospecific, lipophilic	lipophilic	immunospecific
Washing step	+	-	+
Measurement of dye efficiency	-	-	-
Measuring the efficiency of the rinsing step	-	-	-
Stability and intensity of the fluorophore	PE - bright, rapidly photobleached; CellMask -bright, stable for 4 h	CellMask -bright, stable for 4 h	AF488, AF647, AF555 – relatively bright and stable fluorophores
Brightness threshold setting	set on 5 on Nanosight	sensitivity 90, shutter 100, min Brightness 25, frame rate 30 fps (two frames)	no information
Dye concentration	only relative dilution stated, no absolute units; optimized on HCC-derived EVs but not in target serum sample	only relative dilution stated, no absolute units, no information about optimalization	adjusted according to the manufacturer’s instructions
Temperature	overnight at 4°C with agitation for antibodies; 2 h, RT for CellMask	at RT for 1 h in dark	adjusted according to the manufacturer’s instructions
EV purification method	direct labeling of serum, SEC after labeling	TFF, SEC	precipitation
Compounds co-isolated with EVs	lipoproteins, protein aggregates	protein aggregates	lipoproteins, protein aggregates
Assessment of background influence	no information about dye-only, buffer-only controls; staining optimization only on isolated EVs and not directly in serum	no information about dye-only, buffer-only controls	no information about antibody-only, buffer-only controls; immunoaffinity control of an EV-free plasma sample is lacking
Sample concentration	adjusted to 10^4^–10^8^ particles/mL	adjusted to 1 × 10^10^ particles/mL	adjusted according to the manufacturer’s instructions
Precipitation and centrifugation step during EV preparation	-	+	+
Anticipated Labeling specificity	high for immunolabeling, low for CellMask	low	high
Reported pre-analytical variables	no information about serum collection variables (centrifugation steps, time between blood draw and serum preparation, etc.)	+	no information about plasma collection variables (type of anti-coagulant, centrifugation steps, time between blood draw and plasma preparation, etc.)
Impact on functionality of EVs	not tested	not tested	not tested

## 6 Study limitations

EV characterization studies are a rapidly growing field, with our understanding of EV biology expanding every year. This study addresses key aspects of fluorescent EV staining for analysis on currently available instruments and discussed in recent publications, although these factors may evolve with the development of new, more sensitive tools capable of easily distinguishing EVs from background and EV detection in biological samples. In addition, our study does not cover non-fluorescent labeling methods, which are also used in EV characterization and are discussed in detail in the Imanbekova study (Imanbekova et al., 2022).

## 7 Summary

Since the recognition that EVs may be promising non-invasive disease indicators, the scientific community has made enormous efforts to develop EV-specific biomarkers for routine clinical use. Diverse high-resolution techniques for single-vesicle analysis have been developed. Soon it has become clear that fluorescent labeling of specific EV markers may be the only way to discriminate EVs in complex, heterogeneous samples and to quantitatively evaluate specific EV populations, often present only in extremely low abundance, for diagnostic and prognostic purposes. Advanced instruments using fluorescence to analyze EVs, such as f-NTA and nFC, allow researchers to gain insights into EVs properties, with each method providing slightly different information.

A wide spectrum of compounds with fluorescent properties have been developed and used to label vesicles. The choice of an appropriate compound should be made consciously because each type has its advantages and disadvantages, allowing for the discovery of different EV properties. Dyes are often not specific only to EVs; the staining efficiency varies greatly depending on the source and composition of EVs, and some compounds may affect the biological functionality of the vesicles. Regardless of the staining methodology and analysis instrument used, appropriate controls are indispensable. Whenever possible, dye removal or dilution steps should be incorporated. A suitable dye removal method is often dye- and EV-specific and must be chosen based on the sample, anticipated EV recovery, quality ratio, and subsequent downstream EV analysis (Rautaniemi et al., 2021).

Furthermore, analysis should be performed with the awareness of the presence of lipoproteins in biological fluids, which are sometimes challenging to distinguish from EVs. One should also consider the impact of the PC of EVs, which affects their properties and may provide additional EV cargo and completely different, additional biological functions.

Currently, there is an increasing number of publications utilizing EV staining for clinical purposes, including their use as biomarkers. Therefore, it is important for the authors of such studies to be aware of the limitations of the used instruments staining protocols and dyes. This will ensure that their results can be properly interpreted and will have true clinical value in expanding our understanding of EV biology.
